# ﻿Two new endemic species, *Peucedanummiroense* and *P.tongkangense* (Apiaceae), from Korea

**DOI:** 10.3897/phytokeys.210.86067

**Published:** 2022-09-29

**Authors:** Kyeonghee Kim, Hwa-Jung Suh, Jun-Ho Song

**Affiliations:** 1 Plant Resources Division, National Institute of Biological Resources, Incheon 22689, Republic of Korea National Institute of Biological Resources Incheon Republic of Korea; 2 Department of Biology, Daejeon University, Daejeon 34520, Republic of Korea Daejeon University Daejeon Republic of Korea; 3 Herbal Medicine Resources Research Center, Korea Institute of Oriental Medicine, Naju 58245, Republic of Korea Korea Institute of Oriental Medicine Naju Republic of Korea; 4 Department of Biology, Chungbuk National University, Cheongju 28644, Republic of Korea Chungbuk National University Cheongju Republic of Korea

**Keywords:** Apiaceae, FE-SEM, Korea, new species, *Peucedanum*, taxonomy

## Abstract

Two new species of *Peucedanum* (Apiaceae), *P.miroense* and *P.tongkangense*, from Gangwon Province, South Korea, are described. Both species are most similar to *P.elegans* and *P.hakuunense* because of their linear ultimate leaf segments. *Peucedanummiroense* was found on crevices of rocks in mountain summits and can be distinguished by its pubescent ovary, purple anthers, oblong schizocarp, and 1 or (2) vittae per vallecula and 4 on the commissural face. *Peucedanumtongkangense* was found in open areas on rocky cliffs along the Donggang River and can be distinguished by its glabrous ovary, whitish-yellow anthers, narrowly ellipsoid schizocarp, and 3 vittae per vallecula and 4 on the commissural face. Distinguishing characteristics, full descriptions, illustrations, photographs, taxonomic notes on geographical distribution, ecology, and phenology of the two species are presented. An identification key for all Korean species of *Peucedanum* is also provided. In addition, the mericarp surface of two new species and their close relatives are compared using micromorphological analysis.

## ﻿Introduction

*Peucedanum* L. (Apiaceae), represented by 100–120 species, is broadly distributed in the Old World (Hiroe 1979; Pimenov and Leonov 1993; Ostroumova and Pimenov 1997a, b; Shneyer et al. 2003). *Peucedanum* is distinguished from other genera by its dorsally compressed mericarps with narrowly winged marginal ribs and filiform dorsal ribs (Drude 1898; Sheh and Watson 2005). The leaves are ternately or pinnately compound, and the leaflets are variously lobed and pinnatifid, pinnatisect, or sometimes simply toothed (Thellung 1926; Shishkin 1951; Shan and Sheh 1992). The ultimate leaf segments of *Peucedanum* are linear, lanceolate, oblong, or oblanceolate (Thellung 1926; Shishkin 1951; Shan and Sheh 1992).

*Peucedanum* is a complex and heterogeneous genus because many species present a complicated morphological variation of key characters, such as composition of leaves and ribs of mericarps (Pimenov and Leonov 1993; Shneyer et al. 2003). Therefore, many authors have tried to divide the genus *Peucedanum* into several genera, according to the species morphology. For example, Pimenov (1986) newly defined a genus *Kitagawia* Pimenov based on the shapes of mericarps, ribs on mericarps, apex of petals, and morphology of the base of the leaves. Other than genus *Kitagawia*, there are several segregate genera part of *Peucedanum* s.l.; these are *Imperatoria* L., *Thysselinum* Adans., *Cervaria* Wolf, *Holandrea* Reduron, Charpin & Pimenov, and *Leutea* Pimenov (Spalik et al. 2004). However, the generic delimitation and synapomorphy of each group is not clear. Therefore, a comprehensive study including further molecular phylogenetic research with more various species is necessary to understand the phylogenetic relationships among groups of *Peucedanum* s.l.

Some species of *Peucedanum* have been used in traditional medicine for the treatment of various conditions, including coughs, cramps, pain, rheumatism, asthma, angina, and headaches (Morioka et al. 2004; Sarkhail, 2014). The most representative phytochemicals isolated from *Peucedanum* are coumarin compounds called pyranocoumarins and furanocoumarins, which have pharmacological effects in ailments including asthma, rheumatism, and gastrointestinal disorders (Sarkhail 2014; Gurbuz et al. 2018). For example, *P.praeruptorum* Dunn, which is distributed in China, is regarded as an effective medicinal plant with anti-inflammatory, anti- asthmatic, and anti-osteoclastogenic properties (Lee et al. 2015; Song et al. 2015).

To date, nine taxa, including three endemic species, in four sections of *Peucedanum* have been reported in Korea (Chung 1957; Kitagawa 1972; Lee 1996; Park et al. 2017; Lee 2018; Kim et al. 2019). During recent fieldwork and as a part of comprehensive systematic studies of *Peucedanum* in South Korea, we identified two distinct undescribed species of *Peucedanum* that have not been reported previously. One species was on the top of mountains in Gangwon Province and the other was on rocky slopes near rivers in Gangwon and North Chungcheong provinces. After detailed morphological, anatomical, and carpological analysis, we concluded that these plants differed from all other species of *Peucedanum*, particularly from *P.elegans* Kom. and *P.hakuunense* Nakai, which have similar ultimate leaf segments. We therefore propose the name *P.miroense* K. Kim, H.J.Suh & J.H.Song for the plants from the top of the mountains and *P.tongkangense* K. Kim, H.J.Suh & J.H.Song for those from the rocky slopes near the rivers and describe them here. We also present illustrations, a taxonomic key to all species of *Peucedanum* in Korea, images and a map of their distribution.

## ﻿Materials and methods

### ﻿Morphological description

The morphological descriptions of the two new species were based on observation of living plants and specimens collected from the type localities in 2021. We also examined specimens in the herbaria KB, KH, KIOM, and SNU (Thiers 2022) to compare them with related species. Type and voucher specimens were deposited in the Korean Herbarium of Standard Resources, Korean Institute of Oriental Medicine (KIOM). Measurements of morphological structures were performed using a digital vernier caliper (CD-15CP; Mitutoyo, Kawasaki, Japan). Digital images of floral parts were captured by using an Olympus SZX16 stereomicroscope (SM: Olympus, Tokyo, Japan), equipped with an attached Olympus DP21 digital camera (Olympus, Tokyo, Japan). Quantitative data of floral structures obtained from SM images were determined using Digimizer software (version 5.4.3; MedCalc Software, Mariakerke, Belgium).

### ﻿Micromorphological observation

We also observed and compared micromorphological details of the mericarp surface of *Peucedanummiroense* and *P.tongkangense* and their close relatives, *P.hakuunense* and *P.elegans*. The dried mericarps of the four species were rehydrated overnight in a wetting agent (Agepon: distilled water, 1:200) (Agfa Gevaert, Leverkusen, Germany). Rehydrated materials were dehydrated through an ethanol series (50%, 70%, 90%, 95%, and 100%) at room temperature for one hour each. The dehydrated material was immersed in liquid CO_2_ for CPD (SPI-13200JE-AB; SPI Supplies, West Chester, PA, USA) and subsequently mounted on aluminum stubs using a double-sided adhesive conductive carbon disk (05073-BA; SPI Supplies, West Chester, PA, USA). All samples were gold-coated using an ion-sputtering device (208HR; Cressington Scientific Instruments Ltd., Watford, UK) and observed using a low-voltage field-emission scanning electron microscope (FE-SEM: JSM-7600F; JEOL, Tokyo, Japan) at an accelerating voltage of 3–5 kV and a working distance of 8 mm.

## ﻿Taxonomy

### ﻿ Peucedanum miroense

Taxon classificationPlantaeApialesApiaceae

K. Kim, H.J.Suh & J.H.Song sp. nov.

21FDD014-11EA-56D6-B55B-E2AC8AA05AEB

urn:lsid:ipni.org:names:77305892-1

Figs 1 , 2 , 6A

#### Type.

Korea. Gangwon Province: Samcheok-si, Miro-myeon, Naemiro-ri, Swinŭm-san, crevices of rocks on mountain summits, 37°26'37.7"N, 129°01'49.4"E, alt. 540 m, 7 September 2021, *J.H.Song* & *S. Yang*, *KIOM-2021-646-1* [Holotype: KIOM! (Fig. 6A); Isotype KB!].

**Figure 1.**
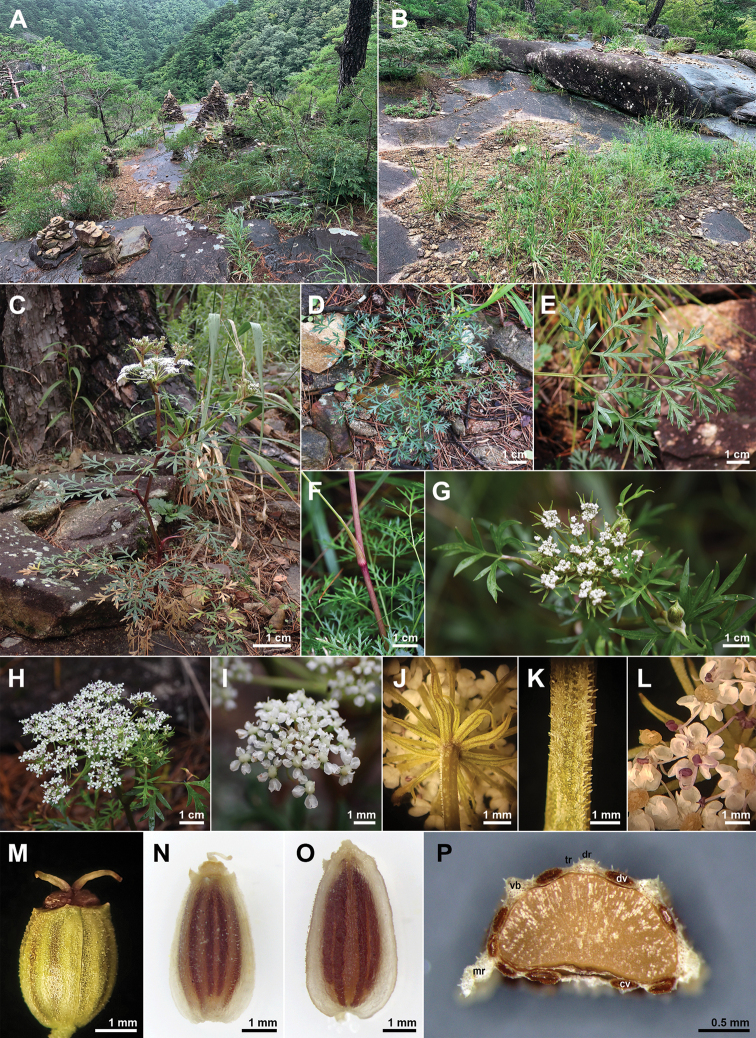
Photographs of *Peucedanummiroense* K. Kim, H.J.Suh & J.H.Song **A–C** habitat **D** basal leaves **E** cauline leaf **F** sheath **G** compound umbel (early flowering stage) **H** compound umbel (mature flowering stage) **I** umbellet (after anthesis) **J** bractlets **K** rays **L** flowers **M** calyx teeth and stylopodium (mature fruiting stage) **N** dorsal side of mericarp **O** commissural side of mericarp **P** transverse plane of mericarp. cv, commissure vittae; dr, dorsal ribs; dv, vallecula vittae; mr, marginal ribs; tr, trichomes; vb, vascular bundles.

**Figure 2.**
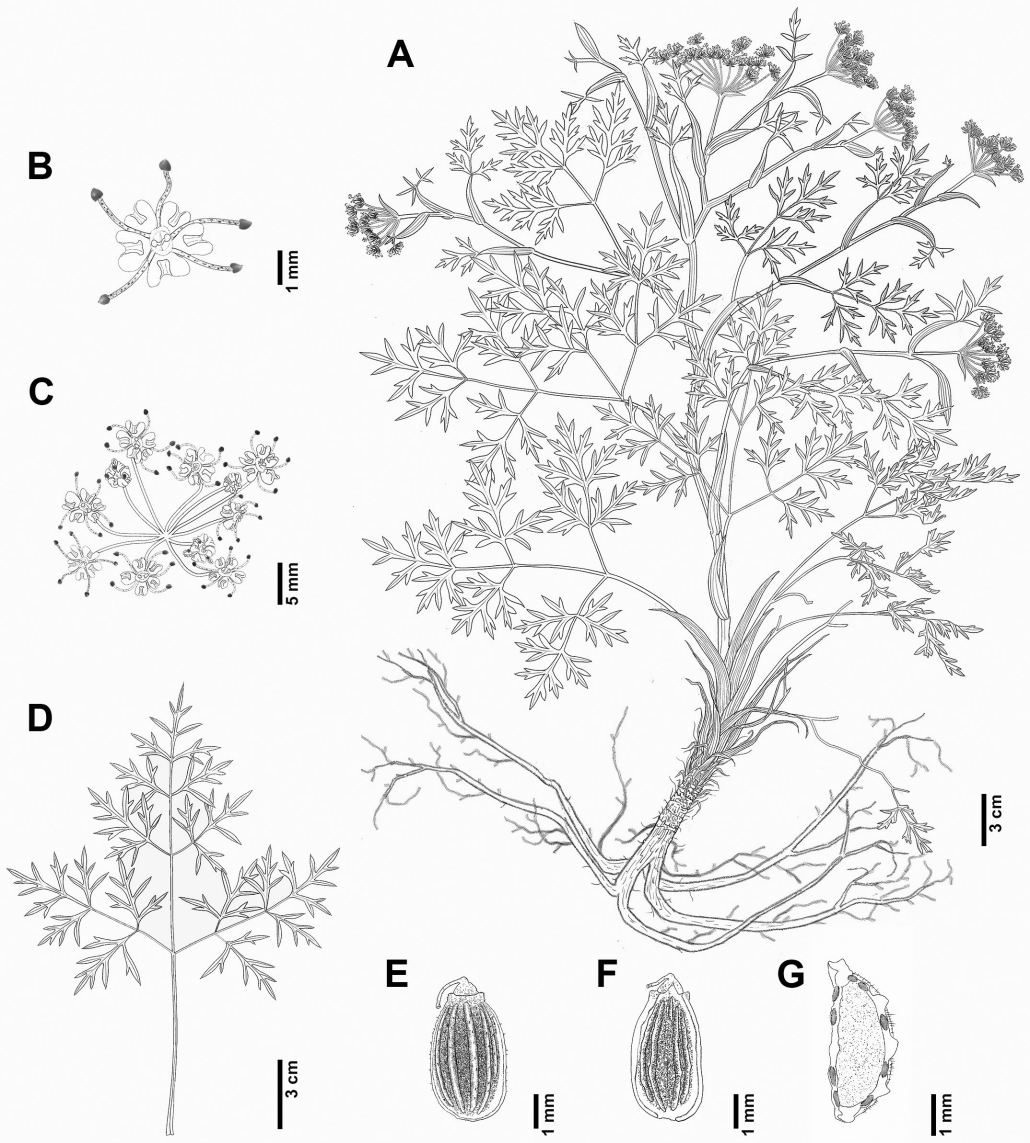
*Peucedanummiroense* K. Kim, H.J.Suh & J.H.Song **A** habit **B** flower **C** umbellet **D** basal cauline leaf **E** mericarp (dorsal side) **F** mericarp (commissural side) **G** mericarp (cross-section) (**A–F***J.H.Song & S. Yang, KIOM2021-646-2*).

#### Diagnosis.

*Peucedanummiroense* is similar to *P.elegans* but differs in its height at anthesis 37–50 cm tall (vs. 60–90 cm) and number of vittae, 8 or 9 vittae (vs. 6), 1 or (2) per vallecula (vs. 1 per vallecula), and 4 on commissure (2 on commissure). *Peucedanummiroense* is similar to *P.hakuunense* in ultimate leaf segments but has 2- or 3-pinnate leaves (vs. 1- or 2-ternate leaves) (Table 1).

**Table 1.** Comparison of major morphological characteristics of *Peucedanummiroense*, *P.tongkangense*, and their close relatives *P.hakuunense* and *P.elegans*.

	*P.miroense*	*P.tongkangense*	P.hakuunense ^*^	P.elegans ^*^
Habitat	crevices of rocks on mountain summits	open areas on rocky cliffs along the river	grassy places on mountain summits	mountain slopes
Height (cm)	37–50	60–120	30–75	60–90
Stems				
Pith	solid	solid	solid	hollow
Branch	branched	much branched	much branched	simple or branched in upper part
Basal leaves				
Division	2-pinnate	3-pinnate	2-ternate	3-pinnate
Persistence	usually deciduous	usually deciduous	persistent	persistent
Outline of blade	ovate to triangular	elliptic to rhombic	triangular to pentagonal	ovate or ovate-oblong
Central/terminal leaflet division	1- or 2-pinnatisect	2-pinnatisect	2-pinnatisect	1-pinnatisect
Central/terminal leaflet shape	triangular or ovate-rhombic	triangular or ovate-rhombic	ovate or rhombic	ovate-rhombic
Ultimate segments shape	narrowly oblong-lanceolate to linear	narrowly oblong-lanceolate to linear	narrowly lanceolate	linear
Ultimate segments apex	acute	acute	acute	cuspidate with spine 1–1.5 mm long
Cauline leaves				
Division	1-pinnatisect	1- or 2-pinnatisect	deeply 3-lobed	entire or 3-lobed
Uppermost one shape	ovate to rhombic	ovate to rhombic	ovate	linear or lanceolate
Inflorescences				
No. of flowers per umbellet	16–23	15–25	15–20	20–24
No. of rays	12–16	16–18	10–20	15–25
No. of bracts	1 or 2	1	usually absent, rarely 1 or 2	5–7
No. of bractlets	6–10	5–6	6 or 8, rarely 9	6–9
Flowers				
Petal color	white	white	white or pinkish	white or pinkish white
Petal shape	obcordate	obcordate	oblong to obovate	obovate-orbicular
Petal size (mm)	0.9–1.2 × 0.7–1.2	0.7–1.3 × 0.9–1.6	approximately 1 × 0.8	0.5–1 × 0.7–1
Anther color	purple	yellowish white	pale yellow	pale yellow
Anther size (mm)	0.3–0.5 × 0.4–0.5	0.5–1.1 × 0.8–1.2	0.2–0.3 × ca. 0.2	0.3–0.4 × 0.2–0.3
Fruit				
Carpophore length (mm)	3.4–4.5	2.1–2.4	2.5–4.5	2.9–3.8
Mericarp size (mm)	3.7–5× 2.4–2.7	3.8–4.4 × 1.5–2	3.7–4 × 2.3–2.5	3–4 × 2–3
Pubescence on dorsal side	moderately to densely pubescent with short simple unicellular hairs	subglabrous to sparsely tuberculate	sparsely tuberculate^†^	moderately to densely pubescent with short simple unicellular hairs^†^
Marginal wings width (mm)	0.2–0.7	0.2–0.3	approximately 0.5	0.5–0.8
No. of vittae	8 or 9	13–16	18–28	6
No. of vittae per vallecula / on commissure	1 or (2) / 4	3 / 4	3 or 4 / 6–12	1 / 2

* Refer to the Park et al. (2017), Flora of Korea, Vol. 5c. Rosidae: Rhamnaceae to Apiaceae. **^†^** Newly updated description of mericarp surface in the present study.

#### Description.

Herbs, perennial, hermaphroditic, 37–50 cm tall. Root a taproot, whitish to pale yellow, elongated, thickened, approximately 20 × 0.6–1.2 cm. Rhizomes erect or ascending, yellowish white, cylindrical, 0.3–1 cm in diameter, woody. Stems erect, purplish below middle, purplish green apically, branched, 4–7 mm in diameter, terete, longitudinally grooved, solid, glabrous, with fibrous remnants of basal leaves. Leaves basal and cauline, alternate, pinnately compound, petiolate, petiole sheathing at base; stipules absent. Basal leaves many, 2-pinnate, usually deciduous; petiole 5.6–9.5 cm long, glabrous; sheath purplish or purplish green, cylindrical, not inflated, 1.1–1.8 cm × 5–7.5 mm, margins scarious, glabrous; blade ovate to triangular in outline, 6.5–11.5 × 7.3–10.6 cm, both surfaces green, glabrous; petiolule of terminal leaflet (0.8–)1.7(–3.5) cm long; terminal leaflet triangular or ovate-rhombic, 1- or 2-pinnatisect, 1.5–2.2 × 1.5–2.6 cm, apex acute, base cuneate, margins entire; petiolule of basal lateral leaflets 0.7–2.7 cm long; lateral leaflets elliptic-ovate to ovate, 1- or 2-pinnatisect, 1.8–5 × 1.3–3.7 cm, apex acute, base cuneate, margins entire, uppermost ones sessile; ultimate segments narrowly oblong-lanceolate to linear, 0.5–1.2 cm × 1.8–3.5 mm. Cauline leaves similar to basal ones and becoming smaller upward; petiole of lower cauline leaves (1.5–)4.8–8 cm long, reduced upward, glabrous; blade elliptic to ovate in outline; uppermost cauline leaves ovate to rhombic, 1-pinnatisect, 0.6–1 × 0.5–1.2 cm, sessile. Inflorescences terminal and lateral, with 2–10 compound umbels, more or less flat-topped, 6.5–7 cm in diameter; umbellets hermaphroditic, 16- to 23-flowered, 1.1–1.5 cm in diameter; peduncle 2.5–6 cm long, sparsely pubescent with short simple unicellular hairs, uppermost part densely pubescent; rays 12–16, spreading to ascending, 1–2.7 cm long, unequal in length, adaxial surface sparsely pubescent with short simple unicellular hairs; bracts 1 or 2, persistent or sometimes caducous, lanceolate, entire, 0.9–1.2 cm × 1–1.8 mm, apex acute, margins scarious, glabrous; pedicels 1.5–7 mm long, adaxial surface sparsely pubescent with simple unicellular hairs; bractlets 6–10, persistent, linear, entire, 2.6–6.7 × 0.4–0.6 mm, apex acute, glabrous. Flowers bisexual, actinomorphic, 1.8–2.1 mm in diameter; calyx 5-toothed; calyx teeth minute, narrowly triangular, 0.2–0.5 × 0.1–0.3 mm, adaxial surface glabrous, abaxial surface sparsely pubescent with short conical simple unicellular hairs; petals 5, white, obcordate, 0.9–1.2 × 0.7–1.2 mm, apex incurved, base cuneate to caudate, with greenish yellow line on abaxial surface, glabrous; stamens 5, alternating with petals, with purplish dots; filaments filiform, 1.2–2 mm long; anthers 2-locular, purple, introrse, versatile, dehiscing longitudinally, subglobose, 0.3–0.5 × 0.4–0.5 mm; pistil 1, 2-carpellate; ovary inferior, syncarpous, 2-locular, moderately to densely pubescent with short simple unicellular hairs; stylopodium conical; styles 2, free, ascending, 0.2–0.5 mm at anthesis, 1.0–1.5 mm in fruit, swollen at base to form a stylopodium, reflexed in fruit; ovule 1 per locule, anatropous, pendulous. Fruit a dry schizocarp composed of 2 mericarps, pale brown to brown at maturity, oblong; carpophore 3.4–4.5 mm long, 2-cleft; mericarps splitting apart at maturity, oblong, dorsally compressed, 3.7–5.0 × 2.4–2.7 mm, moderately to densely pubescent with short simple unicellular hairs on dorsal surface, glabrous on commissural surface; dorsal ribs 3, prominent, not winged; marginal ribs 2, slightly winged; wings 0.2–0.7 mm wide, scarious; secondary ribs absent; vittae (oil tubes) 8 or 9, 1 or (2) per vallecula and 4 on commissure; commissure 1.7–3.6 mm wide. Seed 1 per mericarp; narrowly oblong in cross-section; face plane.

#### Phenology.

Flowering September to October. Fruiting October to November.

#### Etymology.

The specific epithet ‘*miroense*’ refers to Miro-myeon, Samcheok-si, where the type specimen was collected.

#### Vernacular name.

Mi-ro-gi-reum-na-mul.

#### Distribution and ecology.

*Peucedanummiroense* is restricted to only two populations on the summits of Swinŭm-san and Duta-san at Miro-myeon, Samcheok-si, Gangwon Province, South Korea. The two populations are connected to each other. The plants occur in rocky areas at the top of the mountains at an elevation of 540–680 m (Fig. 5). One population, at the type locality on Swinŭm-san, was growing with *Alliumthunbergii* G. Don (Amaryllidaceae), *Dendranthemaboreale* (Makino) Y. Ling ex Kitam. (Asteraceae), *Fraxinussieboldiana* Blume (Oleaceae), *Lespedezabicolor* Turcz., *L.maximowiczii* C.K. Schneid. (Fabaceae), *Peucedanumterebinthaceum* (Fischer ex Trevir.) Turcz. (Apiaceae), *Pinusdensiflora* Siebold & Zucc. (Pinaceae), *Quercusmongolica* Fisch. ex Turcz (Fagaceae), *Rhododendronmucronulatum* Turcz. (Ericaceae), *Sedumpolytrichoides* Hemsl. (Crassulaceae), and *Spodiopogonsibiricus* Trin. (Poaceae). The other population of *P.miroense* on Duta-san was growing with *Aconogononmicrocarpum* (Kitag.) H. Hara (Polygonaceae), *Chrysanthemumzawadskii* Herbich (Asteraceae), and *Geraniumkoreanum* Kom. (Geraniaceae). Each population of *P.miroense* comprised approximately 120 individuals.

#### Additional specimens examined (Paratypes).

Korea. Gangwon Province: Samcheok-si, Miro-myeon, Naemiro-ri, Swinŭm-san, 37°26'46.5"N, 129°01'41.0"E, alt. 535 m, 12 October 2014, *K. Kim & H.-J. Suh, KK#4* (SNU).

#### Proposed IUCN conservation status.

After conducting fieldwork throughout the country and examining specimens from several domestic herbaria, we found out that *Peucedanummiroense* is known only from Miro-myeon, Gangwon. Therefore, according to the IUCN criteria, *P.miroense* is classified as endangered (IUCN 2022; EN D) because the known number of individuals occurring at Swinŭm-san and Duta-san in Gangwon Province, South Korea, is less than 250.

#### Taxonomic notes.

*Peucedanummiroense* is morphologically similar to *P.elegans* and *P.hakuunense* among species with linear ultimate leaf segments. *Peucedanummiroense* is clearly distinguishable from *P.elegans*, which is restricted to mountain slopes in North Korea, by the shape of the leaf apex, the number of bracts, pubescence of the mericarp, and the number of vittae per mericarp (non-overlapping character states). *Peucedanummiroense* has an acute leaf apex, 1 or 2 bracts, moderate to dense pubescence with short simple unicellular hairs on the dorsal surface of the mericarps, and 8 or 9 vittae [1 or (2) per vallecula and 4 per commissure] whereas *P.elegans* has spine-tipped ultimate leaf segments, 5–7 bracts, glabrous mericarps, and 6 vittae (1 per vallecula and 2 per commissure) (Table 1).

Additionally, *P.miroense* is easily distinguishable from *P.hakuunense*, which is only in the southern part of South Korea, on the basis of its 2-pinnate leaves, obcordate petals, purple anthers, 8 or 9 vittae [1 or (2) per vallecula and 4 per commissure]; *P.hakuunense* has 3-ternate leaves, persistent basal leaves, oblong to obovate petals, and 18–28 vittae (3 or 4 per vallecula and 6–12 per commissure) (Table 1).

The natural habitat of *P.miroense* on Swinŭm-san and Duta-san in Gangwon Province is one of the major limestone areas in Korea, with sedimentary rock outcrops consisting of calcium carbonate. *Peucedanummiroense* can be considered a calciphile and added to the limestone flora of Korea (Kim et al. 2021).

### ﻿ Peucedanum tongkangense

Taxon classificationPlantaeApialesApiaceae

K. Kim, H.J.Suh & J.H.Song sp. nov.

F877DB25-3CB1-5FC7-9C0A-D9906B5FB2D8

urn:lsid:ipni.org:names:77305893-1

Figs 3 , 4 , 6B

#### Type.

Korea. Gangwon Province: Jeongseon-gun, Sindong-eup, Unchi-ri, Donggang River, rocky cliffs along the riverside, 37°16'25.7"N, 128°36'33.8"E, alt. 264 m, 8 September 2021, *J.H.Song* & *S. Yang*, *KIOM-2021-802-1* [Holotype: KIOM! (Fig. 6B); Isotype KB!].

#### Diagnosis.

*Peucedanumtongkangense* is similar to *P.miroense*, but differs in its subglabrous (vs. pubescent) ovary, yellowish white (vs. purple) anthers, narrowly ellipsoid (vs. oblong) schizocarp, 13–16 vittae (3 per vallecula, 4 on commissure) [vs. 8 or 9 vittae, 1 or (2) per vallecula, 4 on commissure] per mericarp. *Peucedanumtongkangense* is also similar to *P.elegans* and *P.hakuunense* but is distinct from both in the acute (vs. spine-tipped) apex of the ultimate leaf segments and 2-pinnate (vs. 1- or 2-ternate) leaves (Table 1).

#### Description.

Herb, perennial, hermaphroditic, (60–)75–95(–120) cm tall. Root a taproot, whitish or pale yellow, elongated, thickened, 17–23 × 0.4–1.5 cm. Rhizomes erect or ascending, yellowish white, cylindrical, approximately 0.6–1.1 cm in diameter, woody. Stems erect, purplish green, much branched, 3–9 mm in diameter, terete, longitudinally grooved, solid, glabrous, with fibrous remnants of basal leaves. Leaves basal and cauline, alternate, pinnately compound, petiolate; petiole sheathing at base; stipules absent. Basal leaves many, 3-pinnate, usually deciduous; petiole 8.5–10.5 cm long, glabrous; sheath purplish or yellowish green, cylindrical, not inflated, 1.3–2 cm × 3.6–8.5 mm, margins scarious, glabrous; blade elliptic to rhombic in outline, 15–21.5 × 12–16.8 cm, both surfaces green, glabrous; petiolule of terminal leaflet 2.7–4.8 cm long; terminal leaflet triangular or ovate-rhombic, 2-pinnatisect, 3.5–5 × 2.8–4.1 cm, apex acute, base cuneate, margins entire; petiolule of basal lateral leaflets 1.8–3.8 cm long; lateral leaflets elliptic to elliptic-ovate, 3-pinnatisect, 7.1–9.9 × 4.7–5.4 cm, apex acute, base cuneate, margins entire, uppermost leaflets sessile; ultimate segments narrowly oblong-lanceolate to linear, 1.3–2 cm × 2.8–4.3 mm. Cauline leaves similar to basal ones and becoming smaller upward; petiole of lower cauline leaves (2–)2.8–4.5 cm long, reduced upward, glabrous; blade elliptic to ovate in outline; uppermost cauline leaves ovate to rhombic, 1- or 2-pinnatisect, 0.9–2.4 × 1.1–2.7 cm, sessile. Inflorescences terminal and lateral, with 15–48 compound umbels, more or less flat-topped, 3.5–8.8 cm in diameter; umbellets hermaphroditic, 15- to 25-flowered, 0.5–1.2 cm in diameter; peduncle 2.5–5 cm long, glabrous; rays 16–18, spreading to ascending, 1–2.5 cm long, unequal in length, adaxial surface sparsely pubescent with short simple unicellular hairs; bract 1, persistent or sometimes caducous, lanceolate, entire, 0.7–2 cm × 1–1.5 mm, apex acute, margins scarious, glabrous; pedicels 1.5–2.5(–5) mm long, adaxial surface sparsely pubescent with simple unicellular hairs; bractlets 5–6, persistent, linear, entire, 2.5–7 × 0.4–0.8 mm, apex acute, glabrous. Flowers bisexual, actinomorphic, 2.4–3.2 mm in diameter; calyx 5-toothed; calyx teeth minute, narrowly triangular, 0.2–0.4 × 0.1–0.2 mm, adaxial surface glabrous, abaxial surface sparsely pubescent with short conical simple unicellular hairs or glabrous; petals 5, white, obcordate, 0.7–1.3 × 0.9–1.6 mm, apex incurved, base cuneate to caudate, glabrous; stamens 5, alternating with petals; filaments filiform, 1.6–2.5 mm long; anthers 2-locular, yellowish white, introrse, versatile, dehiscing longitudinally, subglobose, 0.5–1.1 × 0.8–1.2 mm; pistil 1, 2-carpellate; ovary inferior, syncarpous, 2-locular, subglabrous; stylopodium conical; styles 2, free, ascending, 0.3–0.7 mm at anthesis, 1.0–1.7 mm in fruit, swollen at base to form a stylopodium, reflexed in fruit; ovule 1 per locule, anatropous, pendulous. Fruit a dry schizocarp composed of 2 mericarps, pale brown to brown at maturity, narrowly ellipsoid; carpophore 2.1–2.4 mm long, 2-cleft; mericarps splitting apart at maturity, narrowly ellipsoid, slightly dorsally compressed, 3.8–4.4 × 1.5–2 mm, subglabrous to sparsely tuberculate on dorsal side, glabrous on commissural side; dorsal ribs 3, filiform, not winged; marginal ribs 2, slightly winged; wings 0.2–0.3 mm wide, scarious; secondary ribs absent; vittae 13–16, 3 per vallecula and 4 on commissure; commissure 0.9–1.2 mm wide. Seed 1 per mericarp; oblong in cross-section; face plane.

**Figure 3.**
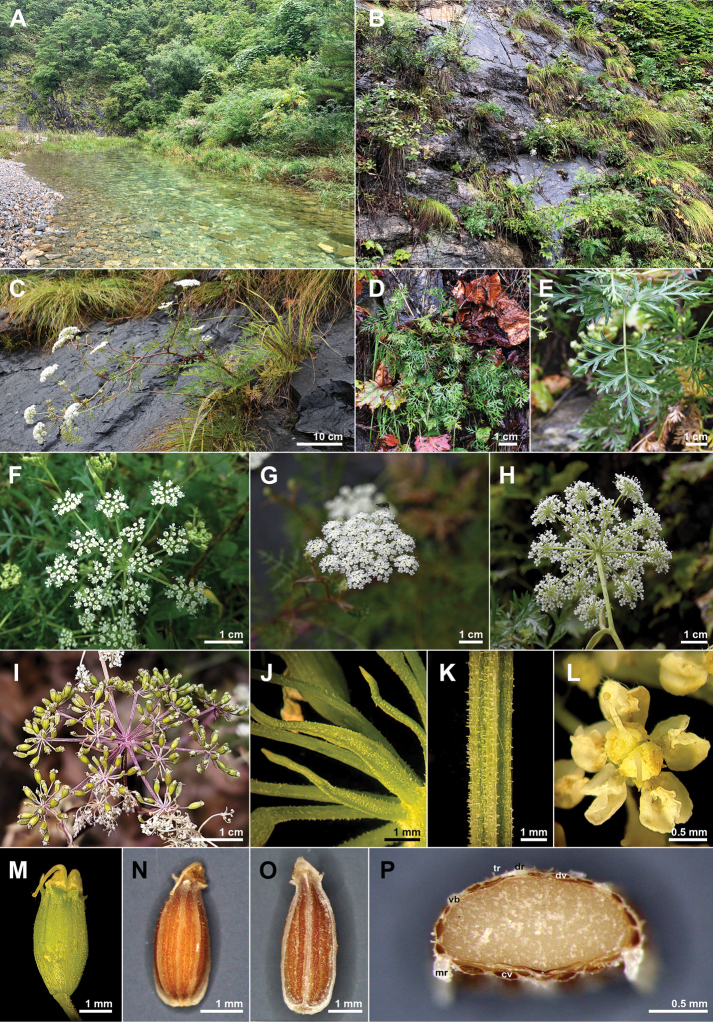
Photographs of *Peucedanumtongkangense* K. Kim, H.J.Suh & J.H.Song **A–C** habitat **D** basal leaves **E** cauline leaf **F** compound umbel (early flowering stage) **G–H** compound umbel (mature flowering stage) **I** compound umbel (fruiting stage) **J** bractlets **K** rays **L** flowers **M** calyx teeth and stylopodium (mature fruiting stage) **N** dorsal side of mericarp **O** commissural side of mericarp **P** transverse plane of mericarp. cv, commissure vittae; dr, dorsal ribs; dv, vallecula vittae; mr, marginal ribs; tr, trichomes; vb, vascular bundles.

**Figure 4.**
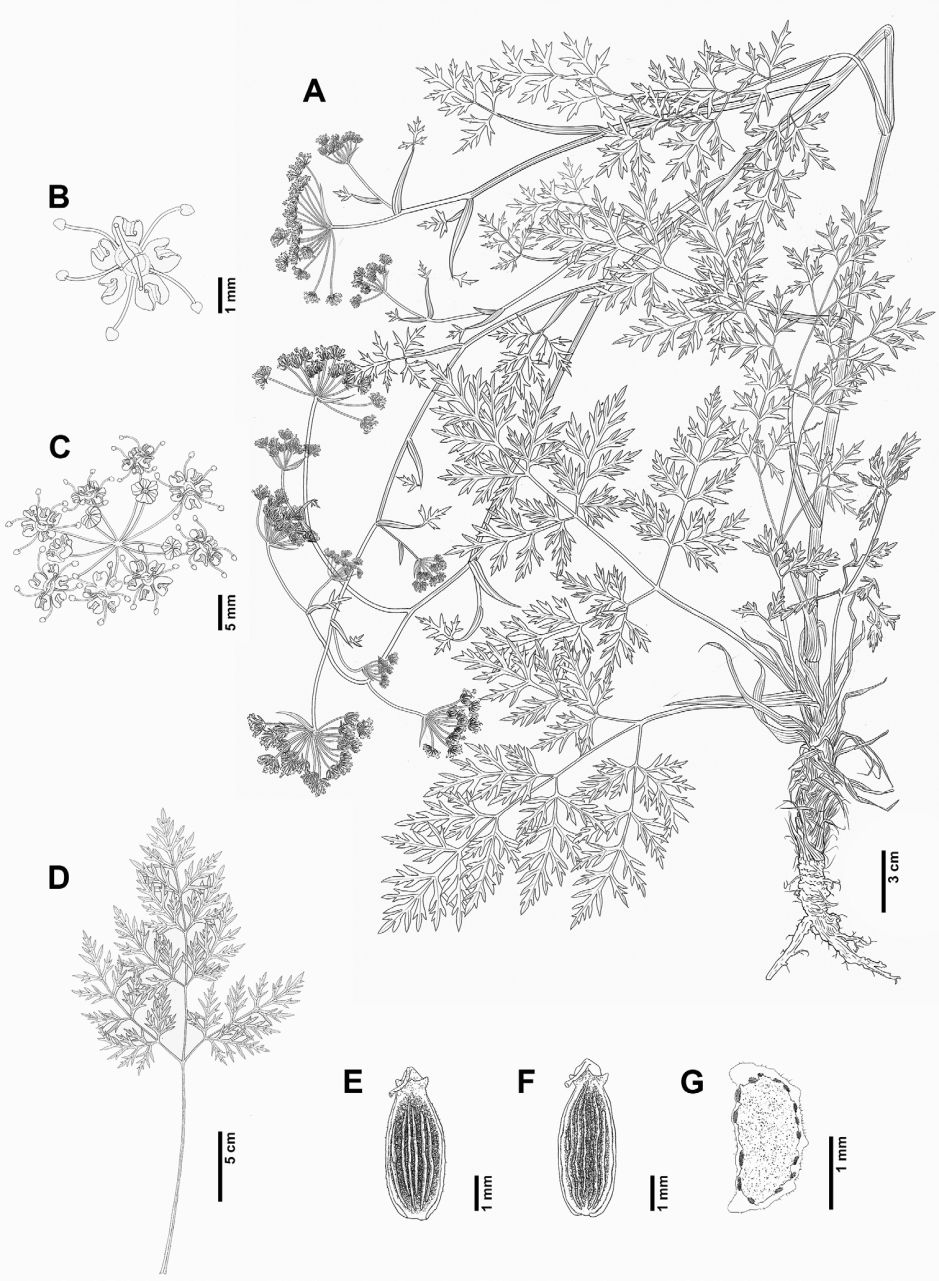
*Peucedanumtongkangense* K. Kim, H.J.Suh & J.H.Song **A** habit **B** flower **C** umbellet **D** basal cauline leaf **E** mericarp (dorsal side) **F** mericarp (commissural side) **G** mericarp (cross-section) (**A–F***J.H.Song & S. Yang, KIOM2021-729-1*).

**Figure 5.**
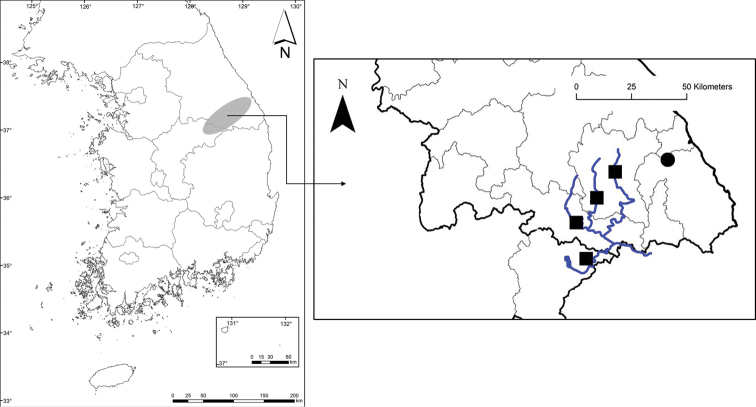
Distribution of *Peucedanummiroense* and *P.tongkangense* = gray ellipse. *P.miroense* = black circle. *P.tongkangense* = black squares. Blue lines: rivers.

**Figure 6.**
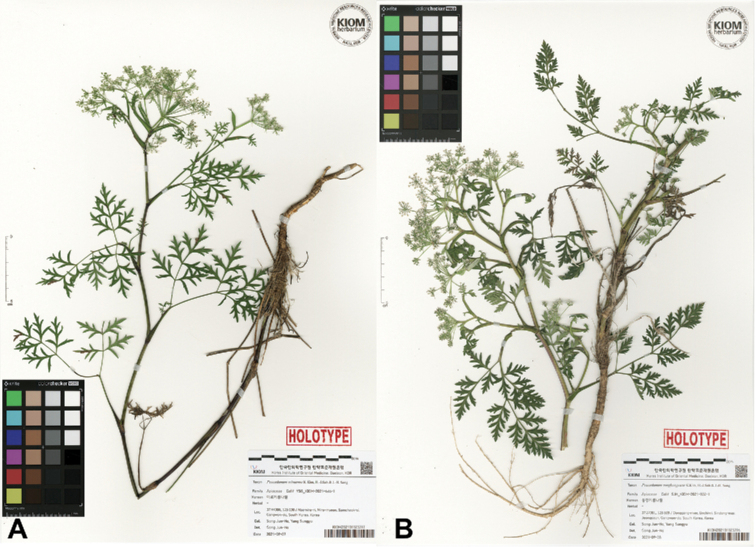
Holotype of **A***Peucedanummiroense* K. Kim, H.J.Suh & J.H.Song (*J.H.Song & S. Yang, KIOM-2021-646-1*) and **B***P.tongkangense* K. Kim, H.J.Suh & J.H.Song (*J.H.Song & S. Yang, KIOM-2021-802-1*).

#### Phenology.

Flowering September to October. Fruiting October to November.

#### Etymology.

The specific epithet ‘*tongkangense*’ refers to the rocky cliffs along the Donggang River, where the type specimen was collected.

#### Vernacular name.

Dong-gang-gi-reum-na-mul

#### Distribution and ecology.

*Peucedanumtongkangense* grows in open areas on rocky cliffs near the Donggang River in Gangwon Province and the Namhangang River in North Chungcheong Province, South Korea. Five populations were found: the type locality and those at Unchi-ri, Sindong-eup, Jeongseon-gun, Gangwon Province, along the Dong-gang river at 150–400 m elevations (Fig. 5). The type locality was growing with ArtemisiasacrorumLedeb.var.iwayomogi (Kitam.) M.S. Park & G.Y. Chung, *Asterscaber* Thunb., *Galinsogaciliata* (Raf.) S.F. Blake (Asteraceae), *Boehmeriaspicata* (Thunb.) Thunb. (Urticaceae), *Calamagrostispurpurea* (Trin.) Trin. (Poaceae), *Carexsiderosticta* Hance (Cyperaceae), *Humulusscandens* (Lour.) Merr. (Cannabaceae), *Isodoninflexus* (Thunb.) Kudô (Lamiaceae), *Parthenocissustricuspidata* (Siebold & Zucc.) Planch. (Vitaceae), *Polystichumcraspedosorum* (Maxim.) Diels (Dryopteridaceae), *Rubiaargyi* (H. Lév. & Vaniot) H. Hara ex Lauener & D.K. Ferguson (Rubiaceae), *Scabiosacomosa* Fisch. ex Roem. & Schult. (Caprifoliaceae), and *Spiraeablumei* G. Don (Rosaceae). Three populations of *P.tongkangense* were also found along the Donggang River where they were growing with *Asteryomena* (Kitam.) Honda (Asteraceae), *Clematisserratifolia* Rehder (Ranunculaceae), and *Trichophorumdioicum* J. Jung & H.K. Choi (Cyperaceae). The fifth population was near the Namhangang River in North Chungcheong Province where it was growing with *Gypsophilaoldhamiana* Miq. (Caryophyllaceae), *Mukdeniarossii* (Oliv.) Koidz. (Saxifragaceae), *Patriniarupestris* (Pall.) Dufr. (Caprifoliaceae), *Potentilladickinsii* Franch. & Sav. (Rosaceae), *Pyrrosiapetiolosa* (Christ) Ching (Polypodiaceae), and *Selaginellastauntoniana* Spring (Selaginellaceae).

#### Additional specimens examined (Paratypes).

Korea. Gangwon Province: Yeongwol-gun, Seo-myeon, Ongjeong-ri, 37°13'5.3"N, 128°20'56.6"E, alt. 234 m, 13 October 2010, *B.-Y. Lee et al.*, *SHY2322* (KB); Gangwon Province: Jeongseon-gun, Hwaam-myeon, Bukdong-ri, 37°22'4.78"N, 128°47'54.35"E, alt. 687 m, 25 September 2012, *G.-H. Nam & J.-H. Kim*, *SHY3-2023* (KB); Jeongseon-eup, Yeotan-ri, 37°22'05.6"N, 128°43'41.9"E, alt. 30 October 2016, *K. Kim & H.-J. Suh*, *KK3510* (SNU); Gangwon Province: Jeongseon-gun, Nam-myeon, Nakdong-ri, 37°18' 38.47" N, 128°42'43.27"E, alt. 719 m, 01 September 2016, *J.-H. Kim & H.-J. Park*, *Beaki161681* (KB).

**Figure 7.**
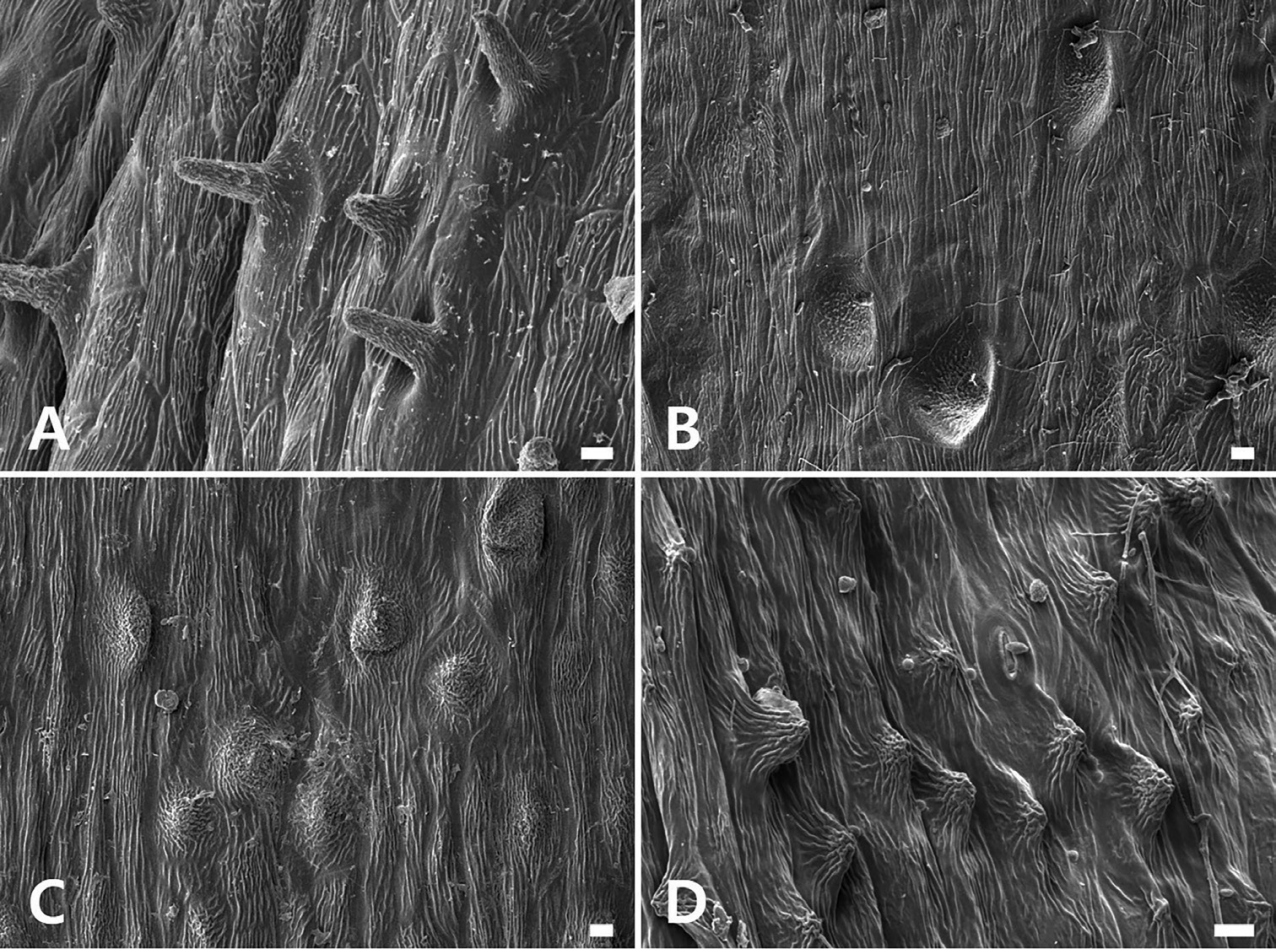
Scanning electron micrographs of mericarp surface of four species of *Peucedanum***A***P.miroense***B***P.tongkangense***C***P.hakuunense***D***P.elegans*. All scale bars: 10 μm.

#### Proposed IUCN conservation status.

After conducting field surveys throughout the country and examining specimens from several domestic herbaria, three more populations along the Donggang River, Gangwon Province were documented for *Peucedanumtongkangense*. According to the IUCN criteria, *P.tongkangense* is classified as least concern (IUCN 2022; LC), because it is distributed widely and a considerable number of individuals is known.

#### Taxonomic notes.

*Peucedanumtongkangense* is morphologically similar to *P.miroense* but it is clearly distinct due to its subglabrous ovary, yellowish white anthers, narrowly ellipsoid schizocarp, and 13–16 vittae (3 per vallecula and 4 on commissure) in mericarp; *P.miroense* has a pubescent ovary, purple anthers, oblong schizorcarp, and 8–10 vittae [1 or (2) per vallecula and 4 on commissure]. Additionally, *P.tongkangense* is similar to *P.elegans* and *P.hakuunense* but is distinguished from *P.elegans* by the acute apex of the ultimate leaf segments (vs. spine-tipped) and from *P.hakuunense* by its 2-pinnate leaves (vs. 1- or 2-ternate leaves) (Table 1).

North Chungcheong Province is also a major limestone area in Gangwon Province. Thus, it is necessary to add *P.tongkangense* to the limestone flora list for Korea (Kim et al. 2021).

##### ﻿Comparative mericarp micromorphology

Micromorphological characteristics of fruits using scanning electron microscopy (SEM) have provided valuable information in classifying and identifying taxa of Apiaceae (Ostroumova 2018 and references therein). Significantly, SEM micrographs helped to visualize trichome types and small rounded projections, such as tubercules (Ostroumova 2018; Lee et al. 2018).

In our study, we found that *P.miroense* and *P.elegans* have short, simple unicellular hairs with a striate surface. Hair length in *P.miroense* was up to 40 μm long, and up to 10 μm long in *P.elegans* (Fig. 7). *Peucedanumtongkangense* and *P.hakuunense* had tubercules 20–30 μm in diameter (Fig. 7). The micromorphological measurements of the mericarps of *P.miroense* and *P.tongkangense* differ from other species. *Peucedanum* has at least two types of mericarp surface.

##### ﻿Key to the species of *Peucedanum* in Korea

**Table:** 

1	Basal and cauline leaves 1- to 3-pinnately compound	**2**
2	Ultimate segments of leaves linear	**3**
3	Umbellets 20- to 44-flowered. Vittae 6, 1 per vallecula and 2 on commissural face	**4**
4	Leaves 2-pinnately compound; blade triangular to broadly ovate in outline; ultimate segments linear-lanceolate, apex acute, not spine-tipped. Bracts 1 or 2	**1. *P.paishanense***
4'	Leaves 2- or 3-pinnately compound; blade ovate in outline; ultimate segments linear, apex spine-tipped. Bracts 5–7	**2. *P.elegans***
3'	Umbellets 16- to 20-(to 27)-flowered. Vittae 8–18, 1–3 per vallecula, 4 or 6 on commissural face	**5**
5	Plants 10–20 cm tall. Bracts 2–7; bractlets 10–12	**3. *P.coreanum***
5'	Plants 50–80 cm tall. Bract 1 or absent; bractlets 6–10	**6**
6	Anthers purple. Mericarp pubescent with short simple hairs. Schizocarp oblong; vittae 8 or 9, 1 or (2) per vallecula, 4 on commissural face	**4. *P.miroense***
6'	Anthers yellowish white. Mericarp subglabrous to sparsely tuberculate. Schizocarp narrowly ellipsoid; vittae 13–16, 3 per vallecula, 4 on commissural face	**5. *P.tongkangense***
2'	Ultimate segments of leaves lanceolate to elliptic, not linear	**7**
7	Apex of ultimate leaf segments acute; vittae 6, 1 per vallecula and 2 on commissure	**6. *P.terebinthaceum***
7'	Apex of ultimate leaf segments rounded; vittae 20–38; 3 or 4 per vallecula and 8–12 on commissure	**8**
8	Leaf blades ovate to triangular in outline, both surfaces glabrous. Bracts 1–4, lanceolate; bractlets 4–8, lanceolate to narrowly triangular	**7. *P.chujaense***
8'	Leaf blades triangular or broadly triangular in outline, both surfaces sparsely pubescent with short simple hairs along veins. Bracts 1, 2 or absent, lanceolate or narrowly triangular; bractlets 8–10, lanceolate to linear	**8. *P.litorale***
1'	Basal and cauline leaves 1- or 2-ternately compound	**9**
9	Leaves coriaceous, both surfaces glaucous; ultimate leaf segments obovate or elliptic. Calyx teeth obsolete. Seed face slightly concave in cross-section	**9. *P.japonicum***
9'	Leaves not coriaceous, adaxial surface green, abaxial surface pale green; ultimate leaf segments linear. Calyx teeth prominent, triangular. Seed face plane in cross-section	**10. *P.hakuunense***

## Supplementary Material

XML Treatment for Peucedanum miroense

XML Treatment for Peucedanum tongkangense
